# Frequency-Following Responses to Complex Tones at Different Frequencies Reflect Different Source Configurations

**DOI:** 10.3389/fnins.2019.00130

**Published:** 2019-02-26

**Authors:** Xiaochen Zhang, Qin Gong

**Affiliations:** ^1^Department of Biomedical Engineering, School of Medicine, Tsinghua University, Beijing, China; ^2^Research Center of Biomedical Engineering, Graduate School at Shenzhen, Tsinghua University, Shenzhen, China

**Keywords:** frequency-following response (FFR), auditory brainstem response (ABR), EEG source imaging, global field synchronization, functional connectivity

## Abstract

The neural generators of the frequency-following response (FFR), a neural response widely used to study the human auditory system, remain unclear. There is evidence that the balance between cortical and subcortical contributions to the FFR varies with stimulus frequency. In this study, we tried to clarify whether this variation extended to subcortical nuclei at higher stimulus frequencies where cortical sources were inactive. We evoked FFRs, in 17 human listeners with normal hearing (9 female), with three complex tones with missing-fundamentals corresponding to musical tones C4 (262 Hz), E4 (330 Hz), and G4 (393 Hz) presented to left, right, or both ears. Source imaging results confirmed the dominance of subcortical activity underlying both fundamental frequency (F0) and second harmonic (H2) components of the FFR. Importantly, several FFR features (spatial complexity, scalp distributions of spectral strength and inter-trial phase coherence, and functional connectivity patterns) varied systematically with stimulus F0, suggesting an unfixed source configuration. We speculated that the variation of FFR source configuration with stimulus frequency resulted from changing relative contributions of subcortical nuclei. Supportively, topographic comparison between the FFR and the auditory brainstem response (ABR) evoked by clicks revealed that the topography of the F0 component resembled that of the click-ABR at an earlier latency when stimulus F0 was higher and that the topography of the H2 component resembled that of the click-ABR at a nearly fixed latency regardless of stimulus F0, particularly for binaurally evoked FFRs. Possible generation sites of the FFR and implications for future studies were discussed.

## Introduction

The auditory system keeps fine temporal representations of sounds at various levels of the auditory pathway, including subcortical (Langner, 1992) and cortical levels (Nourski and Brugge, 2011; Coffey et al., 2016). In humans, these fine temporal representations can be investigated by a steady-state evoked potential named frequency-following response (FFR; Krishnan, 2007), which can be non-invasively recorded on the scalp (Moushegian et al., 1973). An advantage of the FFR is that it faithfully follows the periodic fluctuations of sound waves, thus providing a window to the internal representations of sounds (Kraus et al., 2017).

Surprisingly, the neural origins of the FFR remain under debate. A conventional view is that the generators of the FFR are entirely restricted to subcortical nuclei (Chandrasekaran and Kraus, 2010). This is supported by evidence from comparisons between scalp and deep recordings in animal models (Smith et al., 1975), from lesion studies in animal models (Smith et al., 1975; Gardi et al., 1979) and in humans with brainstem injury (Sohmer et al., 1977), and recently from source-reconstruction studies (Bidelman, 2015; Zhang and Gong, 2017). Although they all suggest that the FFR has only subcortical sources, the exact generating site is under debate; some suggest the predominant role of inferior colliculus (IC; Smith et al., 1975; Sohmer et al., 1977), while others regard the FFR as representing integrated activity from multiple nuclei including not only IC but also cochlear nucleus (CN), superior olive complex (SOC), and/or lateral lemniscus (LL; Gardi et al., 1979). Another view permits cortical contributions among FFR sources and regards the FFR as a representation of sustained activity from the whole auditory system (Coffey et al., 2016). A predominant FFR component was observed at right auditory cortex for FFRs recorded via magnetoencephalography (MEG; Coffey et al., 2016), which leads to more precise source localization than electroencephalography (EEG; Baillet, 2017). In addition, FFR strength at stimulus F0 correlates with activation level of right but not left auditory cortex (Coffey et al., 2017).

A deeper investigation of the FFR source literature points to the possibility that the configuration of the contributing neuron ensembles to the FFR, or FFR source configuration, may vary as a function of stimulus frequency (Bidelman, 2018). In other words, both cortical and subcortical neuronal ensembles are potential generators of the FFR, but the eventual source configuration varies with stimulus frequency and leads to various neuronal activation patterns. Indeed, early studies that used relatively high-frequency stimuli (tone bursts ≥ 250 Hz; Smith et al., 1975; Sohmer et al., 1977; Gardi et al., 1979) tended to ascribe the neural origins of FFR to the auditory brainstem. We now know that FFRs evoked by speech sounds with a low fundamental frequency (F0 = 98 Hz) contain a cortical component when recorded via MEG (Coffey et al., 2016), though it is small when recorded via EEG (Bidelman, 2018). Converging evidence that FFR simulation had a larger cortical component at lower frequencies (Tichko and Skoe, 2017), that only FFRs in response to slow amplitude modulation rates were modulated by attention (Holmes et al., 2018), and that intracranial recordings at auditory cortices contained FFRs at frequencies as high as 200 Hz (Brugge et al., 2009; Nourski and Brugge, 2011) support an unfixed configuration of neural origins underlying the FFR, with FFRs in response to sounds with lower F0s containing higher-level components.

Although there are multiple potential contributors to the FFR at the subcortical level even when the stimulus frequency is high enough to exclude most cortical contributions (Stillman et al., 1978; Galbraith, 1994), it remains unclear whether the source configuration may vary with stimulus frequency. We hypothesize that the dependence of source configuration on stimulus frequency may extend to FFRs in response to higher stimulus frequencies that reflect neural activity solely from subcortical structures. In this study, we examined whether FFRs to three different F0s had different spatial complexity, different scalp distributions of FFR strength and inter-trial phase coherence, and different patterns of functional connectivity. The fundamental frequency (F0) and the second harmonic (H2) components of the FFR were investigated separately. If our hypothesis was correct, we should observe varying topographic features of the FFR with stimulus F0; and we indeed saw evidence in favor of our hypothesis for both F0 and H2 components of the FFR.

A further question we intended to explore was whether the variation of FFR source configuration with stimulus frequency was due to changing relative contributions of subcortical nuclei. Owing to the poor spatial resolution of EEG source imaging, it was virtually impossible to attribute FFR components to different nuclei precisely. However, since the neural sources of the click-evoked auditory brainstem response (ABR) were well investigated in previous studies, we attempted to acquire indirect converging evidence by comparing scalp topographies of temporal-wave peaks of FFRs to different frequencies with those of the click-ABR at different latencies. If the variation of FFR source configuration with stimulus frequency could be attributed to changing relative contributions of subcortical nuclei, we should observe topographic resemblance between FFRs at higher frequencies and click-ABRs at earlier latencies. We observed different patterns of resemblance to the click-ABR for the F0 and H2 components of the FFR, implying complex mechanisms underlying the variation of FFR source configuration with stimulus frequency.

In this study, we made the complex tones missing-fundamental in order to reduce possible contaminations from the cochlear microphonic potential (CM) so that we could ensure the recorded FFRs were dominantly neural. The stimulus frequencies were deliberately set above 200 Hz, so that the possible cortical sources of the FFR were largely excluded. In addition, we presented the stimuli to the left ear, to the right ear, and to both ears, thus acquiring FFRs elicited by both monaural and binaural stimulations. This protocol enabled us to investigate whether the source configuration of the FFR varied with stimulus frequency for all stimulation sides, and whether it varied in the same manner. From the FFR topographies, we could also check whether stimulation side may affect FFR source configuration. We predicted (1) mirrored topographies for FFRs evoked monaurally on the left and right sides, which also provided mutual replication of results, (2) different scalp distributions for monaurally and binaurally evoked FFRs, or to be more specific, more lateralized scalp distributions for monaurally evoked FFRs, since some of the potential FFR sources respond only to stimulations from one side while others seem to respond equally to stimulations from both sides (Marsh et al., 1974), and (3) possibly different variation of FFR source configuration with stimulus frequency for monaurally and binaurally evoked FFRs.

## Materials and Methods

### Subjects

Seventeen students (nine females; mean ± SD age, 25 ± 4 years) from local universities participated in this study. All subjects were native mandarin Chinese speakers with normal hearing. Pure tone thresholds for both ears were all below 25 dB hearing level at octave frequencies from 250 to 8000 Hz. None of the subjects had a history of neurological or psychiatric diseases. Five subjects had the experience of chorusing and/or playing a musical instrument for more than 6 years, and others had virtually no musical experience. All subjects were paid for their time. All subjects gave written informed consent in accordance with the Declaration of Helsinki. The protocol was approved by the Institutional Review Board at Tsinghua University (IRB00008273).

### Stimuli

**FIGURE 1:**
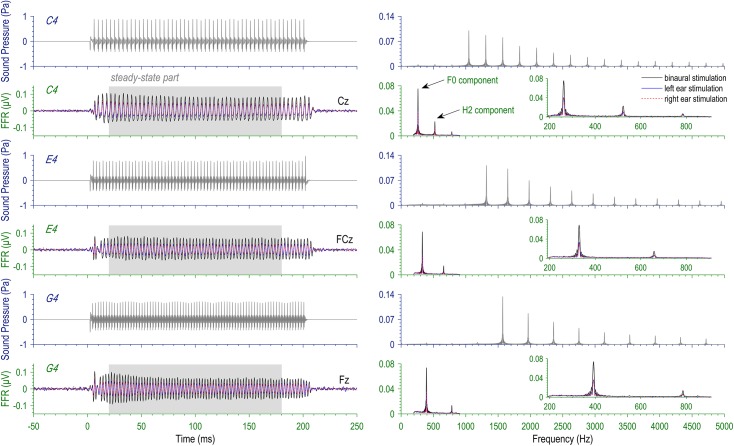
Stimuli and evoked frequency-following responses (FFRs). Three missing-fundamental complex tones at the pitches of musical tones C4 (262 Hz), E4 (330 Hz), and G4 (393 Hz) were presented to the left ear, the right ear, and both ears for each subject (3 × 3 design). Here displayed are the temporal waveforms **(left)** and the spectra **(right)** of the stimuli and the FFRs at typical recording sites. Note that the FFR spectra are based on the steady-state part (20–180 ms).

Three complex tones with missing fundamentals at 262, 330, and 393 Hz were used as stimuli for the FFR session (Figure 1). They were respective frequencies of musical tones C4, E4, and G4. Hereafter, we used C4, E4, and G4 to refer to the three complex tones. The complex tones were synthesized by adding cosine waves at the integer multiples of the fundamental frequency together, with the frequency components at F0 and the second (H2 = 2F0) and the third harmonics (H3 = 3F0) excluded. Exclusion of the first three frequency components rendered all the stimuli missing-fundamental without changing their pitches. The exact F0s of the stimuli were selected out of no specific reasons, but musical pitches seem to be a common choice when FFRs are elicited by complex tones (e.g., Musacchia et al., 2007; Bidelman et al., 2011). Also, in order to reduce contaminations from power-line noise, we avoided the F0s and the 2F0s of stimuli falling on the multiples of 50 Hz. The sound duration was 200 ms, and the sound intensity was calibrated at 80 dB sound pressure level (SPL; with Ear Simulator Type 4157, Brüel and Kjær). Stimuli were delivered to the left ear, to the right ear, or to both ears via a pair of insert earphones (ER-3A; Etymotic Research) under control of STIM2 (Compumedics NeuroScan). The FFR session contained nine blocks; in each block, one type of stimulus was repeatedly presented, monaurally to one ear or binaurally to both, in the same polarity at a rate of 2.4 s^-1^ for at least 2000 trials.

A rarefaction click with a duration of 100 μs was used as stimuli for the click-ABR session. The intensity of the click was calibrated at 97 dB peak-peak equivalent SPL. It was comparable to the intensity of the complex tones used to evoke FFRs in that if the click was extended periodically according to the F0 of any complex tone and was high-pass filtered to exclude the F0, H2, and H3 components, the intensity difference between the complex tone synthesized from the clicks and the actual complex tone was negligible (≤1 dB SPL). Stimulus delivery was the same as in the FFR session. The click was repeatedly presented first to the left ear, then to the right ear, and finally to both ears (or first to the right ear, then to the left ear, and finally to both ears) at a rate of 15 s^-1^ for at least 6000 trials per condition. One subject was excluded due to excessive noise at occipital electrodes.

### Electrophysiological Recording

During recording of electrophysiological data, subjects sat comfortably against an upholstered armchair in an electro-acoustically shielded chamber, and were instructed to minimize movement and avoid chewing or swallowing. Silent films were played for subjects on an iPad (Apple) to keep them awake. EEG data were continuously recorded at a sampling rate of 20 kHz with a SynAmps2 amplifier (gain, 2010; 24-bit; Compumedics NeuroScan) and the software CURRY (7.0.9, Compumedics NeuroScan) from 34 channels (Fp1/2, F3/4/7/8, FC3/4, FT7/8, C3/4, T7/8, CP3/4, TP7/8, P3/4/7/8, PO3/4, O1/2, Fpz, Fz, FCz, Cz, CPz, Pz, POz, Oz) of the international 10/10 system, physically referenced to the midpoint of Cz and CPz, with the ground electrode at AFz, using Quik-Caps (Compumedics NeuroScan). Ag/AgCl electrodes were used, and all impedances were maintained below 10 kΩ during data acquisition.

### Preprocessing

Data pre-processing, including filtering, bad block marking, data segmenting, and averaging, was conducted for each of the conditions (FFR: 3 tones × 3 stimulation sides; click-ABR: 3 stimulation sides) for each subject. The continuously recorded EEG data were first band-pass filtered between 200 and 900 Hz for FFR and between 30 and 1500 Hz for click-ABR, through which ocular artifacts were largely eliminated. The width of the bandpass filter from complete attenuation to complete transmission for the FFR was 40 and 180 Hz for the lower- and upper-limit frequencies, respectively. Hence, no F0 or H2 components of the FFR to be analyzed would be attenuated. Then, data points exceeding ± 50 μV were detected, and the data around them (±200 ms) were marked as “bad blocks” that had been contaminated by myogenic artifacts or electrical noise. For FFR, continuous EEG data were then segmented into 300-ms epochs, which included 50-ms of pre-stimulus data; for click-ABR, they were segmented into 55-ms epochs with 20-ms pre-stimulus data. Only valid epochs without bad block were averaged (mean ± SD epoch number: FFR, 2196 ± 175; click-ABR, 6957 ± 1323). All aforementioned steps of pre-processing and the source imaging were completed in CURRY. Other analyses were realized with custom programs in Matlab. Both averaged and single-trial data would be used later for calculation of FFR measures.

### FFR Source Imaging

In order to examine whether our FFRs were dominated by subcortical but not cortical activity, we applied standardized Low Resolution Electromagnetic Tomography (sLORETA; Pascual-Marqui, 2002) to estimate the current density distribution underlying the FFR, for both F0 and H2 components for all three tones. We used a distributed source model because *a priori* assumption of the number of dipole sources was not available. The advantages of sLORETA are (1) that it is able to realize exact localization without error for simulation of noiseless single source (Pascual-Marqui, 2002), (2) that it is much more sensitive to deep sources, unlike the Minimum Norm (MN) method that favors superficial sources (Pascual-Marqui, 2002), and (3) that it outperforms MN and LORETA with presence of noise (Wagner et al., 2004). The distributed source model contains a dipole, whose orientation and strength are to be determined, in each volume of the whole brain. The grid spacing was set at 5 mm. A pre-computed realistic finite element model (FEM) derived from the MNI (Montreal Neurological Institute) averaged dataset was adopted as the head model. FEM head models provide more precise estimations of the shape and conductivity of the brain than traditional multi-shell spherical head models (Michel et al., 2004). The source imaging was done in the frequency domain for each condition; only grand averaged data were used for source imaging procedure to optimize the precision. Source reconstruction based on Fourier transforms of the response was more suitable for FFR than that based on temporal waveforms, as the response power of FFR concentrated on several discrete frequencies (multiple integers of stimulus F0). The current density reconstructions (CDRs) for both F0 and H2 components for all three tones were displayed on the MNI average brain. Note that the limited spatial resolution of EEG source imaging was very likely to prevent us from distinguishing activations at one nucleus from those at another.

### Calculation of Global Measures of Multi-Channel Data

Global spectral amplitude (GSA) and global field synchronization (GFS) were calculated for each condition for each subject. GSA refers to the variance of responses across all channels in the frequency domain, and GFS refers to the phase consistency of responses across channels; both measures are reference-independent and represent information from multiple channels (Koenig and Pascual-Marqui, 2009). The analysis window was from 20 to 180 ms after stimulus onset to include only the steady-state part of the FFR.

Calculation of both global measures was based on the averaged data. First, the FFR signal in each channel was transformed into the frequency domain with Fourier transform and was represented by a dot on the sine-cosine diagram for each frequency. Therefore, one dataset of the averaged FFR from all 34 channels projected 34 dots to the sine-cosine diagram for each frequency. If there is purely noise at a frequency, the dots will be distributed close to the original point; if there exists an FFR signal at a frequency, the dots will be distributed to a wider range. Therefore, the size of the area of the dot distribution, i.e., the variance of data across channels, could be adopted as a measure of FFR strength. To calculate this measure, a principal component analysis (PCA) was performed to decompose the variation of the data dots, leading to two principal components. The square root of the sum of the two eigenvalues was taken, named GSA, to reflect the inter-channel variation of the FFR data at the frequency under consideration. It measured the strength of multi-channel FFRs in a less biased way than FFR strength calculated in any single channel.

In addition, GFS, defined as the absolute value of the difference between the two eigenvalues normalized by the sum of both eigenvalues, measured spatial complexity in terms of phase synchronization across channels. If the phases of FFRs across channels are highly synchronized, the data dots on the sine-cosine diagram tend to be aligned along a line and the GFS will be large and close to 1. Hence, a high GFS indicates that the multi-channel FFR data are dominated by a single phase. Similarly, when GFS is small, the multi-channel FFR data must be explained by at least two origins with different phases.

The GSA/GFS of the F0 and the H2 component was taken as the mean of GSA/GFS within ±2 Hz around the stimulus F0 and around twice F0, respectively. For each GSA/GFS, the noise floor was estimated within the same frequency range on the averaged GSA/GFS spectrum for the other two stimulus F0s; only spectra for E4 but not those for C4 were used to calculate the noise floor for the GSA/GFS of H2 components evoked by G4, because FFRs evoked by C4 had response power at its H3, which was at the same frequency as the H2 component of FFRs evoked by G4.

### Calculation of Scalp Distribution of FFR Features

Spectral strength and phase coherence were calculated for each channel of the FFR data for each condition for each subject. The analysis window was also from 20 to 180 ms after stimulus onset.

Calculation of spectral strength was based on the averaged data. First, the FFR data were re-referenced to the average of all channels. Then, the spectrum of the FFR signal in each channel was obtained with Fourier transform. The spectral strength of the F0 (or H2) component of the FFR at a certain channel was calculated as the mean of spectral amplitude within ±2 Hz around the stimulus F0 (or twice F0).

Calculation of phase coherence was based on the single-trial data; only valid epochs were included. First, each epoch was re-referenced to the average of all channels, and the single-trial signal in each channel was transformed into the frequency domain via Fourier transform. Then, for each epoch for each channel, the frequency-domain signal was normalized by the magnitude at each frequency so as to preserve only phase information, resulting in a phase vector as a function of frequency. Afterwards, the phase vectors were averaged across epochs and then the moduli of the averaged phase vectors for each channel were taken, named inter-trial phase locking (ITPL), as shown in Equation (1).

(1) $$ITPL^{\left(k\right)}(f)=\left|\frac{1}{N}\sum_{i=1}^{N}\frac{X_{i}^{\left(k\right)}(f)}{\left|X_{i}^{\left(k\right)}(f)\right|}\right|$$

where *ITPL*^(*k*)^ (*f*) refers to the ITPL for the *k*th channel, *N* refers to the number of valid epochs, $X_{i}^{\left(k\right)}$ (*f*) refers to the Fourier transform of the *i*th FFR epoch in the *k*th channel, and |•| refers to the modulus of a complex number. ITPL varies between 0 (not synchronized in phase across trials at all) and 1 (perfectly synchronized in phase across trials). The phase coherence of the F0 (or H2) component of the FFR at a certain channel was calculated as the mean of ITPL within ±2 Hz around the stimulus F0 (or twice F0).

### Topographic Comparison of FFR Features Across Stimulus F0s

Since a fixed configuration of neural sources will not lead to different scalp distributions (i.e., topographies), a change in topography indicates a change in the underlying source configuration. Topographies of FFR spectral strength and phase coherence were compared across the three tones mainly by visual inspection, since there was no applicable test to our knowledge. We also obtained topographies of temporal-wave peaks of the FFR so as to compare FFR scalp distributions across stimulus F0s with topographic analysis of variance (TANOVA), a non-parametric test to detect differences between topographies (Murray et al., 2008). The topography of peaks in the temporal waveform of the FFR evoked by each tone on each stimulation side was determined for each subject, separately derived for F0 and H2 components. First, the multichannel FFRs were band-pass filtered channel by channel within ±4 Hz around the stimulus F0 or twice F0 by a frequency-domain Gaussian filter. Then, the global field power (GFP, standard deviation across all channels) at every time point was calculated as the standard deviation of all the values across the channels at that time point. Peaks of the time course of GFP were identified and the largest 25% of all peaks were preserved in order to ensure good signal quality. Next, topographies at the time points of these preserved peaks were selected and those that were vertex positive (automatically classified with *K*-mean clustering; see Murray et al., 2008) were averaged to obtain the final topography. Afterwards, TANOVAs were performed to examine whether the topography of FFR peaks differed across stimulus F0s.

### Calculation of Functional Connectivity

We explored whether FFR origins varied with stimulus F0 via comparison of functional connectivity patterns across stimulus F0s. Functional connectivity of the FFR was calculated on sensor level for each condition for each subject. It reflected the stability of the phase difference between two sensors. A more stable phase difference between two sensors is an indicator of a stronger flow of information in between, suggesting that these two sensors are more closely connected. The analysis window was also from 20 to 180 ms after stimulus onset.

Calculation of FFR functional connectivity was based on single-trial data. First, twenty epochs were selected randomly from the pool of all valid epochs and were averaged to obtain an estimation of the FFR. This was repeated for 100 times to obtain 100 FFR estimations. Then, for each FFR estimation, cross spectrum was calculated between any two of all the sensors, and for each pair of sensors, the frequency-domain signal was normalized by the magnitude at each frequency so as to preserve only phase information, resulting in a phase vector as a function of frequency. Afterwards, the phase vectors were averaged across estimations and the moduli of the averaged phase vectors for each pair of sensors were taken, named functional connectivity, as shown in Equation (2).

(2) $$FC^{\left(k,l\right)}(f)=\left|\frac{1}{N}\sum_{i=1}^{N}\frac{X_{i}^{\left(k\right)}(f)•{\bar{X}}_{i}^{\left(l\right)}(f)}{\left|X_{i}^{\left(k\right)}(f)|•|X_{i}^{\left(l\right)}(f)\right|}\right|$$

where *FC*^(*k,l*)^ (*f*) refers to the functional connectivity between the *k*th and the *l*th sensors, *N* refers to the number of FFR estimations (here 100), $X_{i}^{\left(k\right)}$ (*f*) and $X_{i}^{\left(l\right)}$ (*f*) refer to the Fourier transforms of the *i*th FFR estimations in the *k*th and the *l*th channels, respectively, and ${\bar{X}}_{i}^{\left(l\right)}$ (*f*) refers to the complex conjugate of $X_{i}^{\left(l\right)}$ (*f*). The eventual functional connectivity between two sensors for the F0 (or H2) component of the FFR was calculated as the mean of functional connectivity within ±2 Hz around the stimulus F0 (or twice F0).

### Comparison of FFR Functional Connectivity Patterns Across Stimulus F0s

The investigation of the patterns of FFR functional connectivity was based on the decomposition of the functional connectivity data and separately conducted for each principal pattern derived from two PCAs performed for F0 and H2 components (Leonardi et al., 2013). First, the functional connectivity data were demeaned. Then, a new set of bases, hereafter referred to as *eigenconnectivity* (EC), was determined to best explain the data variance. As the majority of the data variance can usually be attributed to the first few ECs, others can be discarded for simplification. Since the FFRs involved in both PCAs contained responses to left- and right-ear stimulations, it was reasonable to assume a pair of mutually mirrored ECs. For the F0 component, the retained ECs indeed included such a pair, but this was not the case for the H2 component. Therefore, varimax rotation was applied to the retained ECs for the H2 component, trying to obtain a pair of ECs with mirrored distribution, which turned out to be useful. After the basis change, the demeaned functional connectivity data could be represented as the coordinates, hereafter referred to as *weights*, which represented the differences in functional connectivity pattern of FFR across conditions.

### Topographic Comparison Between FFR and Click-ABR

In order to compare topographically between the FFR and the click-ABR, we calculated a bootstrap-based similarity statistic as a function of time for each tone for each stimulation side, for both F0 and H2 components. The similarity statistic was determined time point by time point. At each time point, the actual global dissimilarity (DISS) was calculated as the GFP of the difference *z*-score map between grand-averaged topographies of FFR peaks and those of the click-ABR at that time point; then all the topographies were pooled together and randomly labeled as “FFR” or “click-ABR,” and the DISS between “FFR” and “click-ABR” was calculated; the previous step was repeated for 5000 times, so as to form a distribution of DISS. The topographic similarity statistic referred to the percentage of the number of DISS values that are higher than the real one. A small statistic suggested a big difference in topography between FFR and click-ABR, while a large statistic suggested similar topographies for FFR and click-ABR.

Apart from the similarity statistic, we also derived the latency when the FFR was most topographically correlated (Pearson’s *r*) with the click-ABR in the time range of 3 and 11 ms for each subject, for each tone, for each stimulation side, and for both F0 and H2 components. The FFR “latency” was compared with the latency of each wave of the click-ABR (waves III, V, VI, and VII), which was determined for each subject as a reference for the latency of highest topographic correlation between the FFR and the click-ABR. To obtain the latencies of ABR waves, first, the GFP as a function of time was calculated for the multichannel click-ABRs. Then, the latency when the time course of GFP reached its largest peak within 4.5 and 7 ms was defined as the latency of wave V; the latency of wave III was determined within a 2.5-ms time range ahead of the latency of wave V; the latency of wave VI was determined within a 2.5-ms time range after the latency of wave V; the latency of wave VII was determined within a 3-ms time range after the latency of wave VI. Since wave V was clear for every subject, only the GFP as a function of time was used for derivation of the latency of wave V. However, for derivation of the latencies of wave III, VI, and VII, when there was no GFP peak within the aforementioned time range, the GFP time course would be replaced during the calculation with a single-channel wave of the click-ABR, average of FCz and Cz referenced to average of TP7 and TP8. Waves I and II were not analyzed, because they did not have sufficient signal-to-noise ratio in every subject.

### Statistical Tests

In this study, repeated measures analyses of variance (RMANOVAs) and paired *t*-tests were used (two-tailed). The number of subjects was always 17 for tests on FFR measures; it was 16 as long as the click-ABR was involved. For RMANOVAs, the number of within-subject factors was declared in Section “Results”; Greenhouse–Geisser correction was applied if sphericity could not be assumed.

## Results

### FFR Strength Measured by Global Spectral Amplitude

**FIGURE 2:**
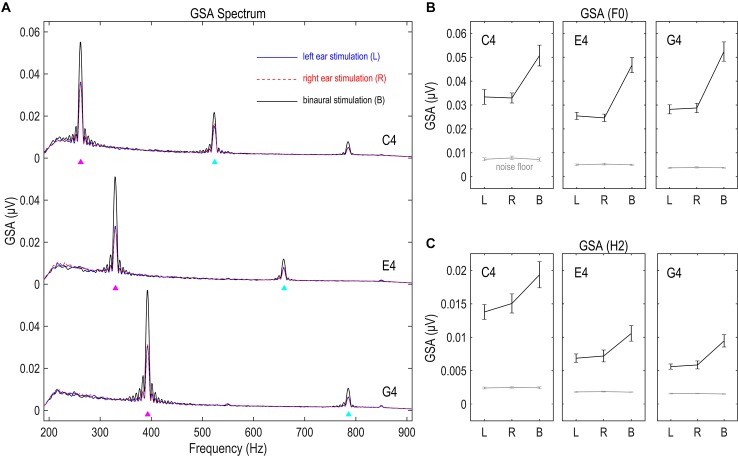
Global spectral amplitude (GSA) of FFR. **(A)** GSA spectra of the FFR. **(B)** GSA of the F0 component. **(C)** GSA of the H2 component. GSA is a reference-independent measure of strength of multichannel FFRs, representing the variance of the Fourier-transformed data across channels. The FFR in response to each stimulus has clear spectral peaks at the fundamental frequency (F0) and the second harmonics (H2). The strength of both F0 and H2 components significantly exceeds noise floor, suggesting good signal quality. Binaural stimulations lead to significantly larger GSA than monaural stimulations, suggesting the capability of this measure in reflecting FFR strength. Triangles point to the frequencies of stimulus F0s and twice F0s. Error bars refer to ±SEM.

The GSA of the FFR had clear spectral peaks at the stimulus F0 and twice F0 for all nine conditions (Figure 2A), suggesting the existence of the F0 and H2 components of the FFR. The GSA of both F0 and H2 components exceeded noise floor to a significant extent for all three tones and all three stimulation sides (df = 16, *t* ≥ 5.877, *p* < 0.001; Figure 2B,C), indicating that the responses recorded in this study were of good quality.

We compared the GSA of FFRs to stimulations on different sides, for both F0 and H2 components and for all three tones. Greater GSA is expected for binaurally evoked FFRs, since binaural stimulations activate neural ensembles to a larger extent than monaural stimulations. Six RMANOVAs with one within-subject factor (stimulation side) were performed on the GSA of the F0 and the H2 components for each stimulus F0. The effect of stimulation side was significant in all six RMANOVAs (*F*_2,32_ ≥ 15.466, *p* < 0.001, η^2^ ≥ 0.492; Figure 2B,C). *Post hoc* pairwise comparisons (with Bonferroni correction, hereafter) showed stronger GSA for binaurally evoked FFRs (vs. left ear, *p* < 0.001; vs. right ear, *p* ≤ 0.003) and similarly strong GSA for FFRs evoked on the left and right sides (*p* ≥ 0.692). These findings indicate that GSA indeed reflected the strength of multi-channel FFR data.

### Source Imaging of FFRs

**FIGURE 3:**
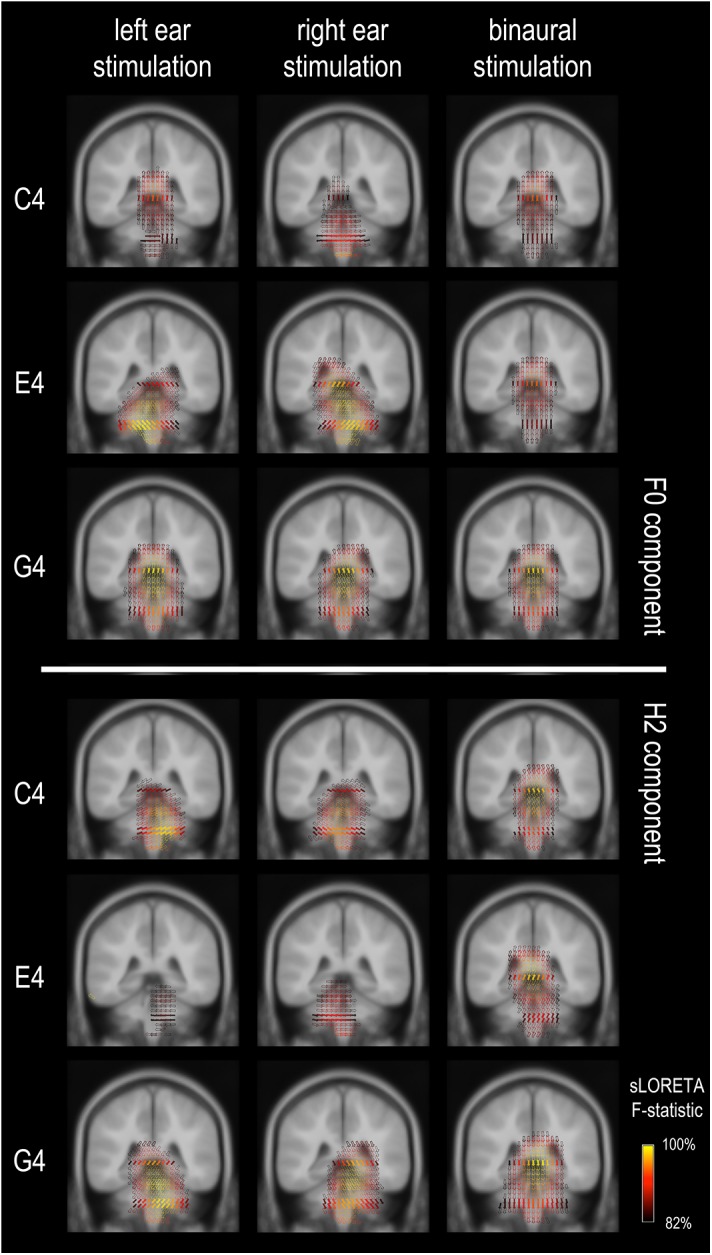
Frequency-following response source imaging. Reconstructed sources of the F0 and H2 components are at subcortical levels, suggesting dominance of subcortical sources for the FFR in response to relatively high stimulus F0s (≥262 Hz). The exact subcortical nuclei from which the FFR originates cannot be determined. The equivalent dipoles (represented by the arrows) point to multiple directions across voxels for the F0 component of the FFR to monaural C4, suggesting high complexity in source configuration. Only sources with a strength larger than 82% of the maximum are displayed; solid arrows refer to the sources exactly on this coronal slice (*y* = –35), whereas hollow ones refer to those nearby (≤3 mm).

We confirmed, by estimating the current density distribution underlying the grand-averaged FFR for both F0 and H2 components for all three tones, that FFRs in this study were dominated by subcortical activity (Figure 3), in line with a previous study using EEG source imaging (Bidelman, 2018). As shown in Figure 3, the strongest activations were located in subcortical regions; there was almost no activation at auditory cortices. However, it was not clearly revealed how the subcortical sources were distributed. In general, the activations were distributed within a broad range, impossible to pinpoint the generation sites precisely. The source orientations tended to be vertical for binaural conditions but oblique for monaural conditions. In addition, the sources for the F0 component to monaural C4 had multiple orientations, while those in other conditions seemed to have a predominant orientation across volumes.

### FFR Spatial Complexity Measured by Global Field Synchronization

**FIGURE 4:**
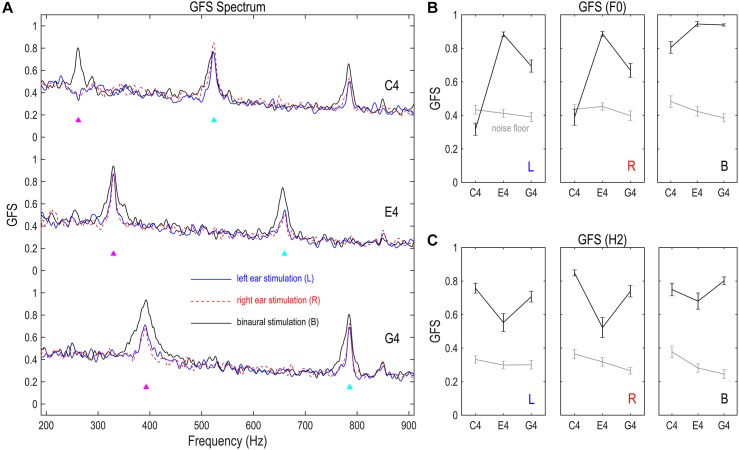
Global field synchronization (GFS) of FFR. **(A)** GFS spectra of the FFR. **(B)** GFS of the F0 component. **(C)** GFS of the H2 component. GFS is a reference- and strength-independent measure of spatial complexity of multichannel FFRs, varying between 1 (perfectly synchronized FFRs across channels) and 0 (completely random response phase across channels). The GFS of both F0 and H2 components significantly exceeds noise floor, except for the F0 component of the FFR monaurally evoked by C4. This indicates a relatively complex source configuration underlying the F0 component of monaurally evoked FFRs to lower stimulus F0s, consistent with the source imaging results. Binaural stimulations lead to lower spatial complexity than monaural stimulations for the F0 component, suggesting possible cancelation between FFR components which renders the sources underlying the F0 component of binaurally evoked FFRs more focal. Importantly, FFR spatial complexity varies with stimulus F0 for the F0 and H2 components of monaurally evoked FFRs and for the F0 component of binaurally evoked FFRs, supporting a varying FFR source configuration with stimulus F0. GFS spectra were smoothed for display. Triangles point to the frequencies of stimulus F0s and twice F0s. GFS of the F0 and H2 components were calculated based on original GFS spectra but not the smoothed ones. Error bars refer to ±SEM.

Spatial complexity of the FFR, or the dominance of a single phase across channels, was measured via GFS (Koenig and Pascual-Marqui, 2009). As shown in Figure 4A, the GFS as a function of frequency had clear peaks at the stimulus F0 for FFRs monaurally evoked by E4 and G4, but not for FFRs monaurally evoked by C4; it had clear peaks at the stimulus F0 for FFRs binaurally evoked by all three tones, and also at twice F0 for all nine conditions. For FFRs in response to C4 on the left side, the GFS of the F0 component was significantly smaller than noise floor (df = 16, *t* = –3.170, *p* = 0.006; Figure 4B). For FFRs in response to C4 on the right side, the GFS of the F0 component was comparable to the noise floor (df = 16, *t* = -0.846, *p* = 0.410; Figure 4B). For the remaining conditions, the GFS of the F0 or H2 component was significantly higher than noise floor (*t* ≥ 3.563, *p* ≤ 0.003; Figure 4B,C). The lower GFS for the F0 component of the FFR monaurally evoked by C4 indicated that the FFRs across channels were not dominated by a single phase; that is to say, a mixture of at least two sources with different phases underlay the F0 component of the FFR to monaural C4. This high spatial complexity might be related to the multiple dipole orientations revealed by source imaging (c.f. section “Source Imaging of FFRs” and Figure 3). In contrast, the GFS for the F0 component of the FFR evoked by E4 was high (mean ± SD: left, 0.883 ± 0.057; right, 0.887 ± 0.063; binaural, 0.945 ± 0.057), suggesting a dominant phase.

The spatial complexity of both F0 and H2 components varied with stimulus F0. We performed two RMANOVAs with two within-subject factors (stimulus F0 and stimulation side) on the GFS of the F0 and the H2 components of monaurally evoked FFRs. For the F0 component, the effect of stimulus F0 was significant (*F*_2,32_ = 76.929, *p* < 0.001, η^2^ = 0.828; Figure 4B). *Post hoc* pairwise comparisons suggested highest GFS for FFRs to E4 (vs. C4 and G4, *p* < 0.001) and significantly higher GFS for FFRs to G4 than for FFRs to C4 (*p* < 0.001). The effect of stimulation side was not significant (*F*_1,16_ = 0.667, *p* = 0.426, η^2^ = 0.040; Figure 4B). Neither was the interaction of stimulus F0 and stimulation side (*F*_2,32_ = 1.518, *p* = 0.235, η^2^ = 0.087; Figure 4B). For the H2 component, the effect of stimulus F0 was also significant (*F*_2,32_ = 13.596, *p* < 0.001, η^2^ = 0.459; Figure 4C). *Post hoc* pairwise comparisons suggested significantly lower GFS for FFRs to E4 (vs. C4, *p* = 0.002; vs. G4, *p* = 0.010) but similar GFS for FFRs to C4 and G4 (*p* = 0.132). However, the effect of stimulation side was not significant (*F*_1,16_ = 1.405, *p* = 0.253, η^2^ = 0.081; Figure 4C). Neither was the interaction of stimulus F0 and stimulation side (*F*_2,32_ = 2.412, *p* = 0.106, η^2^ = 0.131; Figure 4C). We also performed two RMANOVAs with one within-subject factor (stimulus F0) on the GFS of the F0 and H2 components of binaurally evoked FFRs. The effect of stimulus F0 was significant for the F0 component (*F*_2,32_ = 12.997, *p* = 0.001, η^2^ = 0.448; Figure 4B) but not for the H2 component (*F*_2,32_ = 2.821, *p* = 0.074, η^2^ = 0.150; Figure 4C).

To investigate the effect of binaural stimulation, six RMANOVAs with one within-subject factor (stimulation side) were performed on the GFS of the F0 and H2 components for each of the three tones. For the F0 component, binaural stimulation reduced the spatial complexity compared with monaural stimulation (Figure 4B). The effect of stimulation side was significant in all three RMANOVAs (*F*_2,32_ ≥ 8.050, *p* ≤ 0.001, η^2^ ≥ 0.335). *Post hoc* pairwise comparisons suggested significantly higher GFS for FFRs evoked binaurally (vs. left ear, *p* ≤ 0.020; vs. right ear, *p* ≤ 0.008) and similar GFS between FFRs evoked on the left and right sides (*p* ≥ 0.593). Therefore, the F0 component of binaurally evoked FFRs enjoyed less spatial complexity, possibly due to cancelation of components with opposite phases in FFRs evoked by left ear and right ear stimulations. Nevertheless, for the H2 component, binaural stimulation did not reduce the spatial complexity as for the F0 component (Figure 4C). The effect of stimulation side was not significant for E4 (*F*_2,32_ = 3.136, *p* = 0.057, η^2^ = 0.164) or G4 (*F*_2,32_ = 3.303, *p* = 0.066, η^2^ = 0.171). It was significant for C4 (*F*_2,32_ = 5.329, *p* = 0.020, η^2^ = 0.250), but *post hoc* pairwise comparisons suggested significantly higher GFS for FFRs evoked on the right side (vs. left ear, *p* = 0.003; vs. binaural, *p* = 0.030) and similar GFS between FFRs evoked on the left side and FFRs evoked binaurally (*p* = 1.000).

### Scalp Distribution of FFR Features

**FIGURE 5:**
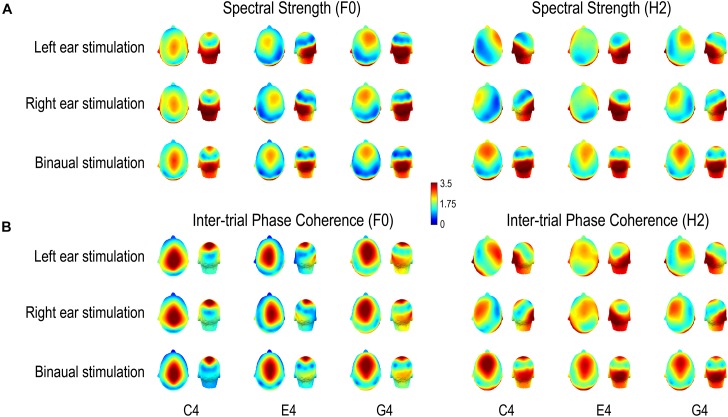
Scalp distributions of **(A)** FFR spectral strength and **(B)** inter-trial phase coherence. Both spectral strength and phase coherence are of large values in two regions, one temporo-occipital and one central-frontal, except that for the F0 component of the FFR to C4, the phase coherence is weak in the temporo-occipital region and for the H2 component of the FFR to monaural E4, the spectral strength is weak in the central-frontal region. Importantly, scalp distributions of both FFR measures vary with stimulus F0 for both F0 and H2 components of FFRs to all three complex tones, supporting a varying FFR source configuration with stimulus F0. For the F0 component, the area where the FFR measures are of large values in the central-frontal region moves from vertex forward to frontal regions with increasing stimulus F0; it stays frontal for the H2 component across stimulus F0s. For both F0 and H2 components, the laterality of the area where the FFR measures are of large values in the temporo-occipital region changes with stimulus F0. Note that normalized data are displayed.

As shown in Figure 5, FFRs evoked on the left side and those evoked on the right side had mirrored topographies; FFR evoked binaurally had topographies symmetrical according to the midline. Both spectral strength and phase coherence of the FFR had relatively large values in two regions, one temporo-occipital and one central-frontal. However, the phase coherence was poor in the temporo-occipital region for the F0 component of FFRs to C4 and the spectral strength was weak in the central-frontal region for the H2 component of FFRs to monaural E4.

The scalp distributions of both spectral strength (Figure 5A) and phase coherence (Figure 5B) varied with stimulus F0. For the F0 component, the area with large values in the central-frontal region tended to move from vertex forward to frontal positions with increasing stimulus F0; in monaural conditions, this area seemed to be along midline for C4, lateralized to the ipsilateral side of stimulation for E4, and lateralized to the contralateral side for G4; in binaural conditions, this area maintained along the midline. On the other hand, the area where the FFR measures were of large values in the temporo-occipital region did not move forward with increasing stimulus F0. It was lateralized to the contralateral side of stimulation for C4 (only for spectral amplitude) and E4 in monaural conditions, lateralized to the ipsilateral side for G4 in monaural conditions, and stayed symmetrical according to the midline for all three tones in binaural conditions. For the H2 component, the area with large values in the central-frontal region tended to stay at frontal positions despite increasing stimulus F0, but it was lateralized to the contralateral side of stimulation for C4 and G4 (to different extents) in monaural conditions and was along the midline for E4 (only for phase coherence) in monaural conditions and for all three tones in binaural conditions. The area with large values in the temporo-occipital region seemed to be lateralized to the ipsilateral side of stimulation for C4 and G4 in monaural conditions, lateralized to the contralateral side for E4 in monaural conditions, and stayed symmetrical according to the midline for all three tones in binaural conditions.

Differences between topographies of peaks in FFR temporal waves across stimulus F0s were statistically examined by TANOVAs. The topographies were determined by averaging vertex-positive maps of the FFR at time points when the GFP reached a positive peak. The topographies of both F0 and H2 components varied with stimulus F0 for each stimulus side (*p* ≤ 0.002; Figure 7B). Together, the maps showing variation with stimulus F0 for both F0 and H2 components of the FFR indicate different source configurations for FFRs in response to sounds at different F0s.

### Functional Connectivity Pattern of FFR

**FIGURE 6:**
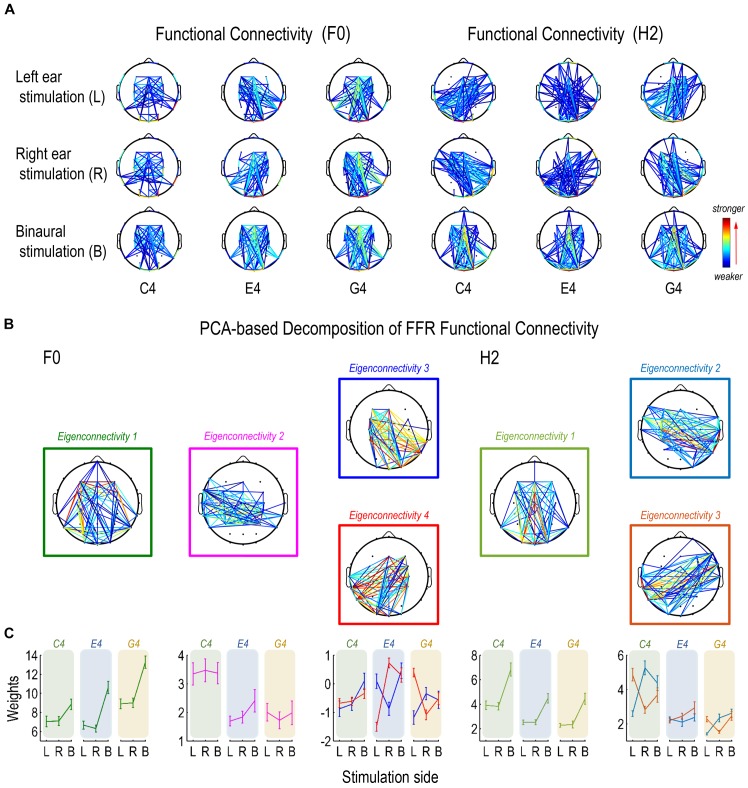
Functional connectivity pattern of FFR. **(A)** Functional connectivity refers to the inter-trial coherence of phase difference between each pair of channels. Functional connectivity patterns of the FFR vary with stimulus F0 for both F0 and H2 components. **(B)** Decomposition of connectivity patterns for the F0 component results in four principal components (eigenconnectivities, ECs), one representing a symmetrical network (EC1), one representing a slightly left lateralized network that is more activated for FFRs to C4 (EC2), and two representing a pair of mirror-distributed lateralized networks (EC3/4). Decomposition of connectivity patterns for the H2 component results in three ECs, one representing a symmetrical network similar as for the F0 component (EC1) and two representing a pair of mirror-distributed networks (EC2/3). **(C)** As to the F0 component, the two lateralized networks (EC3/4) are similarly activated for FFRs to C4; the network lateralized to the contralateral side of stimulation is more activated for FFRs to E4, and the network lateralized to the ipsilateral side is more activated for FFRs to G4. As to the H2 component, the pair of mirror distributed networks (EC2/3) are activated in a similar way across stimulation sides for FFRs to C4 and G4, but not for FFRs to E4. Error bars refer to ±SEM. For display, only 15% pairs of electrodes with the closest connections are displayed in each network.

Patterns of functional connectivity, or the inter-trial coherence of phase difference between any two electrodes, of the FFR varied with stimulus F0, at least for monaurally evoked FFRs (Figure 6A).

We performed two PCAs to investigate the functional connectivity patterns of the F0 and the H2 components of the FFR on sensor level. For the F0 component, the first four ECs were retained. Their eigenvalues were over 0.90 and they explained 68.35% of the entire data variance. For the H2 component, the first three ECs were retained. Their eigenvalues were over 0.70 and they explained 70.03% of the entire data variance. Different ECs corresponded to different patterns of sensor-level connections, which were indirect representations of underlying neural networks comprising various sources.

For the F0 component, the first eigenconnectivity (EC1) represented a symmetrical network (Figure 6B). An RMANOVA with two within-subject factors (stimulus F0 and stimulation side) was performed on the weights of EC1. As shown in Figure 6C, the effect of stimulus F0 (*F*_2,32_ = 32.786, *p* < 0.001, η^2^ = 0.672), the effect of stimulation side (*F*_2,32_ = 188.460, *p* < 0.001, η^2^ = 0.922), and their interaction (*F*_2,32_ = 10.869, *p* < 0.001, η^2^ = 0.405) were significant. As to stimulus F0, *post hoc* pairwise comparisons suggested significantly larger weights for G4 (vs. C4 and E4, *p* < 0.001) but similar weights between C4 and E4 (*p* = 1.000). As to stimulation side, *post hoc* pairwise comparisons suggested significantly larger weights for binaural stimulation (vs. left ear and right ear, *p* < 0.001) but similar weights between left and right ear stimulation (*p* = 1.000).

The second eigenconnectivity (EC2) represented a slightly left-lateralized network with connections between temporal and central electrodes (Figure 6B). An RMANOVA with two within-subject factors (stimulus F0 and stimulation side) was performed on the weights of EC2. Only the effect of stimulus F0 was significant (*F*_2,32_ = 18.506, *p* < 0.001, η^2^ = 0.536; Figure 6C). *Post hoc* pairwise comparisons suggested significantly larger weights for C4 (vs. E4, *p* < 0.001; vs. G4, *p* = 0.002) but similar weights between E4 and G4 (*p* = 1.000). Hence, this network was more activated for C4 and possibly played a vital role for FFRs evoked by sounds at lower F0s.

The third and the fourth eigenconnectivities (EC3/4) represented two lateralized, mutually mirrored networks (Figure 6B). EC3 emphasized connections between right temporo-occipital and left central-frontal electrodes (right-lateralized), while EC4 emphasized connections between left temporo-occipital and right central-frontal electrodes (left-lateralized). Three RMANOVAs with two within-subject factors (stimulation side and EC) were performed on the weights of EC3 and EC4 for each stimulus F0. For FFRs to C4, only the effect of stimulation side was significant (*F*_2,32_ = 20.958, *p* < 0.001, η^2^ = 0.567; Figure 6C). *Post hoc* pairwise comparisons suggested significantly larger weights for binaural stimulation (vs. left and right ear stimulation, *p* < 0.001) but similar weights between left-ear and right-ear stimulation (*p* = 0.576). For FFRs to E4, the interaction of stimulation side and EC was significant (*F*_2,32_ = 36.754, *p* < 0.001, η^2^ = 0.696; Figure 6C). Paired *t*-tests showed that when stimuli were presented to left ear, the weights of EC3 was significantly larger than those of EC4 (df = 16, *t* = 4.630, *p* < 0.001); when stimuli were presented to right ear, the weights of EC3 was significantly smaller than those of EC4 (df = 16, *t* = -5.537, *p* < 0.001); when stimuli were presented binaurally, the weights of the two ECs were similar (df = 16, *t* = 0.578, *p* = 0.572). For FFRs to G4, the interaction of stimulation side and EC was also significant (*F*_2,32_ = 20.578, *p* < 0.001, η^2^ = 0.563; Figure 6C). Paired *t*-tests showed that when stimuli were presented to left ear, the weights of EC3 was significantly smaller than those of EC4 (df = 16, *t* = -4.941, *p* < 0.001); when stimuli were presented to right ear, the weights of EC3 was significantly larger than those of EC4 (df = 16, *t* = 2.495, *p* = 0.024); when stimuli were presented binaurally, the weights of the two ECs were similar (df = 16, *t* = 0.187, *p* = 0.854). Therefore, these two networks were differentially activated by different stimulus F0s, particularly in monaural conditions. For sounds at a lower F0, they were activated to similar extents; as stimulus F0 increased, the network lateralized to the contralateral side of stimulation was more activated than the network lateralized to the ipsilateral side in the first place; but as stimulus F0 further increased, the latter network was more activated than the former one.

For the H2 component, the first eigenconnectivity (EC1) also represented a symmetrical network, similar to EC1 for the F0 component (Figure 6B). An RMANOVA with two within-subject factors (stimulus F0 and stimulation side) was performed on the weights of EC1. As shown in Figure 6C, the effect of stimulus F0 (*F*_2,32_ = 22.369, *p* < 0.001, η^2^ = 0.583), the effect of stimulation side (*F*_2,32_ = 86.556, *p* < 0.001, η^2^ = 0.844), and their interaction (*F*_2,32_ = 4.391, *p* = 0.003, η^2^ = 0.215) were significant. As to stimulus F0, *post hoc* pairwise comparisons suggested significantly larger weights for C4 (vs. E4 and G4, *p* < 0.001) but similar weights between E4 and G4 (*p* = 1.000). As to stimulation side, *post hoc* pairwise comparisons suggested significantly larger weights for binaural stimulation (vs. left ear and right ear, *p* < 0.001) but similar weights between left and right ear stimulation (*p* = 1.000).

The second and the third eigenconnectivities (EC2/3) represented two mutually mirrored networks, but different from EC3/4 for the F0 component (Figure 6B). EC3/4 for the F0 component emphasized vertical channels more, whereas EC2/3 for the H2 component seemed to include horizontal channels. Three RMANOVAs with two within-subject factors (stimulation side and EC) were performed on the weights of EC2 and EC3 for each stimulus F0. For FFRs to C4, the interaction of stimulation side and EC was significant (*F*_2,32_ = 55.275, *p* < 0.001, η^2^ = 0.776; Figure 6C). Paired *t*-tests showed that when stimuli were presented to left ear, the weights of EC2 was significantly smaller than those of EC3 (df = 16, *t* = -8.435, *p* < 0.001); when stimuli were presented to right ear and to both ears, the weights of EC2 was significantly larger than those of EC3 (df = 16; right ear: *t* = 7.455, *p* < 0.001; binaural: *t* = 2.631, *p* = 0.018). For FFRs to E4, the interaction of stimulation side and EC was not significant (*F*_2,32_ = 2.420, *p* = 0.105, η^2^ = 0.131; Figure 6C). The two ECs had significantly different weights (EC2 < EC3, *F*_1,16_ = 4.719, *p* = 0.045, η^2^ = 0.228). The effect of stimulation side was significant (*F*_2,32_ = 3.846, *p* = 0.047, η^2^ = 0.194); *post hoc* pairwise comparisons suggested significantly larger weights for binaural than for right ear stimulations (*p* = 0.037) but similar weights between left ear and right ear stimulations (*p* = 1.000), or between left ear and binaural stimulations (*p* = 0.198). For FFRs to G4, the interaction of stimulation side and EC was significant (*F*_2,32_ = 24.857, *p* < 0.001, η^2^ = 0.608; Figure 6C). Paired *t*-tests showed that when stimuli were presented to left ear, the weights of EC2 was significantly smaller than those of EC3 (df = 16, *t* = -6.809, *p* < 0.001); when stimuli were presented to right ear, the weights of EC2 was significantly larger than those of EC3 (df = 16, *t* = 4.566, *p* < 0.001); when stimuli were presented binaurally, the weights of EC2 and EC3 were similar (df = 16, *t* = 0.962, *p* = 0.350). Therefore, these two networks were differentially activated by different stimulus F0s, particularly under monaural stimulations. For C4 and G4, the network with connections between ipsilateral temporo-occipital and contralateral central-frontal sensors was more activated than the network with connections between contralateral temporo-occipital and ipsilateral central-frontal sensors; but for E4, the two networks seemed to be activated to similar extents.

The decomposition of functional connectivity patterns of the F0 and H2 components of the FFR revealed clear differences across stimulus F0s, indicating differences in the source configuration of FFRs to different tones, at least in monaural conditions. There seemed to be a symmetric network shared by both F0 and H2 components. For FFRs to sounds at lower F0s, a specialized network was activated for the F0 component. Also, the laterality of functional connectivity patterns for both F0 and H2 components varied with stimulus F0.

### Topographic Comparison Between FFR and Click-ABR

**FIGURE 7:**
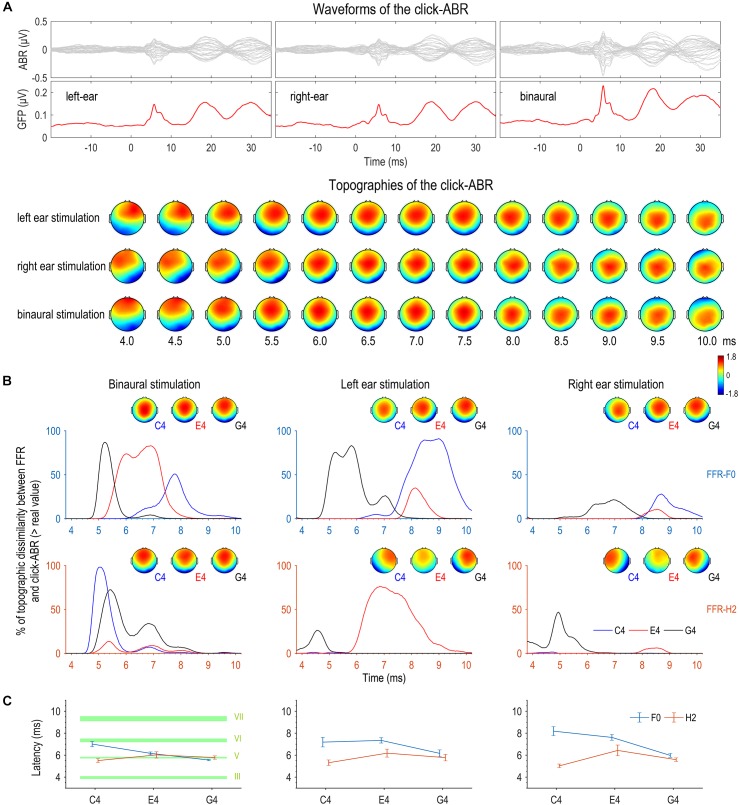
Topographic comparison between FFR and click-ABR. **(A)** Auditory brainstem responses (ABRs) were evoked by clicks with a comparable level to the complex tones used to evoke FFRs. Displayed here are the grand averaged waveforms of the click-ABR across channels, the global field power (GFP) of the click-ABR, and the topographies of the click-ABR as a function of time. Note that the topographies at later latencies correspond to neural activity at higher levels. **(B)** For the F0 component, the bootstrap-based similarity statistic reaches its peak first for G4, later for E4, and latest for C4. For the H2 component, the similarity peaks appear at similar latencies for the three tones in binaural conditions; in monaural conditions, the topographic similarity between the FFR to C4 and the click-ABR is poor within the entire time range corresponding to brainstem origins. **(C)** The results of the topographic similarity statistic and those of individual latencies based on topographic correlations are highly consistent for the F0 and H2 components in binaural conditions. The F0 component of binaural FFRs to a higher stimulus F0 shows the greatest topographic resemblance to the click-ABR at an earlier latency, whereas the H2 component of binaural FFRs to different stimulus F0s shows the greatest topographic resemblance to the click-ABR at similar latencies. Specifically, for the F0 component of the FFR, the latency for C4 is comparable to that of wave VI (generation site: inferior colliculus, IC), the latency for E4 is comparable to that of wave V (superior olive complex, SOC; and/or lateral lemniscus, LL), and the latency for G4 is earlier than that of wave V but later than that of wave III (cochlear nucleus, CN). For the H2 component of the FFR, the latency is comparable to that of wave V (SOC and/or LL) for all three tones. Error bars refer to ±SEM. The upper and lower limits of green shaded rectangles refer to mean ± SEM.

We compared topographies of peaks in FFR temporal waves and topographies of click-ABR to examine whether the FFR to a higher stimulus F0 was topographically similar to a later wave of the click-ABR. The topographies of the click-ABR as a function of time are displayed in Figure 7A. We assumed that a topography of the click-ABR at a later latency reflected neural activity at a higher level.

First, a bootstrap-based statistic representing topographic similarity between FFR and click-ABR was calculated at every time point for each tone for each stimulation side. As shown in Figure 7B, for the F0 component, this similarity statistic reached its peak first for G4, later for E4, and latest for C4. The time courses of similarity statistics for the three tones were most separable for binaural stimulation. For the H2 component, results were more complicated. In monaural conditions, the similarity statistic reached its peak earlier for G4 than for E4, but the FFR to C4 seemed to be topographically correlated to the click-ABR at no time point (Figure 7B). In binaural conditions, the similarity peaks appeared at similar latencies for the three tones, with the similarity peak for C4 slightly earlier (Figure 7B).

In addition, the latency when the click-ABR had the most similar topography to the FFR to each tone presented to each stimulation side was calculated for every subject, for both F0 and H2 components. Six RMANOVAs with one within-subject factor (stimulus F0) were performed on this latency for all stimulation sides for both F0 and H2 components. For the F0 component, the effect of stimulus F0 was not significant in left monaural conditions (*F*_2,30_ = 3.295, *p* = 0.051, η^2^ = 0.180; Figure 7C), but it was significant in right monaural conditions (*F*_2,30_ = 12.895, *p* < 0.001, η^2^ = 0.462; Figure 7C); *post hoc* pairwise comparisons suggested that the latency for the FFR to G4 was earlier (vs. C4, *p* = 0.002; vs. E4, *p* < 0.001) and the latency for the FFR to C4 and E4 was similar (*p* = 0.874). The effect of stimulus F0 was also significant in binaural conditions (*F*_2,30_ = 25.635, *p* < 0.001, η^2^ = 0.631; Figure 7C); *post hoc* pairwise comparisons suggested that the latency for the FFR to G4 was earliest (vs. C4, *p* < 0.001; vs. E4, *p* = 0.001) and the latency for the FFR to E4 was earlier than for the FFR to C4 (*p* = 0.004). For the H2 component, the effect of stimulus F0 was not significant in left monaural (*F*_2,30_ = 1.587, *p* = 0.228, η^2^ = 0.096; Figure 7C) and binaural conditions (*F*_2,30_ = 1.565, *p* = 0.226, η^2^ = 0.094; Figure 7C). It was significant in right monaural conditions (*F*_2,30_ = 4.360, *p* = 0.044, η^2^ = 0.225; Figure 7C), though *post hoc* comparisons revealed no significant difference between any two of the three tones (C4 vs. E4, *p* = 0.052; C4 vs. G4, *p* = 0.093; E4 vs. G4, *p* = 0.525).

The results of the topographic similarity statistic and those of the individual latency based on topographic correlations were highly consistent for the F0 and H2 components in binaural conditions. In monaural conditions, they were only consistent for the F0 component in right monaural conditions. The better consistency in binaural conditions might be resulted from more focal source configurations, which were more akin to that of the click-ABR. Therefore, the F0 component of FFRs binaurally evoked by sounds with higher stimulus F0s may originate from subcortical structures at lower levels, whereas the H2 component of FFRs binaurally evoked by sounds at different F0s may originate from nuclei at similar positions.

We compared the latency when FFR and click-ABR showed highest topographic correlation and the latencies of various waves of the click-ABR (waves III, V, VI, and VII) for binaural conditions. Results are displayed in Table 1 and Figure 7C. For the F0 component of the FFR, the latency for C4 was comparable to that of wave VI, the latency for E4 was comparable to that of wave V, and the latency for G4 was earlier than that of wave V but later than that of wave III. For the H2 component of the FFR, the latencies for C4, E4, and G4 were all comparable to that of wave V.

**Table 1:** Comparison of the latencies of ABR waves and the time point of highest topographical similarity between binaurally evoked frequency-following response (FFR) and click-ABR.

			ABR-III	ABR-V	ABR-VI	ABR-VII
		**95% Confidence Interval (ms)**	**3.743–4.176**	**5.679–5.871**	**7.048–7.646**	**8.883–9.760**

FFR-F0	C4	6.481–7.544	*^∗∗∗^*	*^∗∗^*	*n.s.*	*^∗∗∗^*
	E4	5.876–6.455	*^∗∗∗^*	*n.s.*	*^∗∗∗^*	*^∗∗∗^*
	G4	5.402–5.705	*^∗∗∗^*	*^∗^*	*^∗∗∗^*	*^∗∗∗^*
FFR-H2	C4	5.120–5.905	*^∗∗∗^*	*n.s.*	*^∗∗∗^*	*^∗∗∗^*
	E4	5.400–6.650	*^∗∗∗^*	*n.s.*	*^∗^*	*^∗∗∗^*
	G4	5.447–6.122	*^∗∗∗^*	*n.s.*	*^∗∗∗^*	*^∗∗∗^*


*^∗∗∗^p < 0.001; ^∗∗^p < 0.01; ^∗^p < 0.05; n.s., *p* > 0.05 (Bonferroni corrected)*.

## Discussion

In this study, the source configuration of FFRs to sounds with F0s above 200 Hz was investigated. Previous studies focus on lower stimulus frequencies and documented cortical contributions to the F0 component (Bidelman, 2015, 2018; Coffey et al., 2016) and dominant subcortical contributions to higher frequencies components of the FFR (Bidelman, 2018). Since accumulating evidence supports a varying FFR source configuration as a function of stimulus frequency (Tichko and Skoe, 2017; Holmes et al., 2018), we hypothesize that the source configuration of FFRs to sounds with F0s above 200 Hz is also varying like the FFRs to sounds with lower F0s, and our findings are supportive of this hypothesis.

First, source imaging of the FFRs showed that subcortical sources dominated the FFRs to sounds with a pitch at or above 262 Hz (C4; Figure 3), consistent with previous reports (Bidelman, 2015, 2018; Zhang and Gong, 2017). However, the specific generating sites of the FFR could not be identified, largely owing to the limitations of the sLORETA method in spatial resolution. The sLORETA method may fuse nearby sources with similar orientations (Wagner et al., 2004), which is very likely for FFR source imaging since the potential sources of the FFR are spatially close. We can see that the sources with different orientations are likely to be distinguished (Figure 3; monaural C4), but their exact locations are blurred also. Since it is rather hard to track the source configuration of the FFR with EEG source imaging techniques, further analyses are focused on response topographies.

Since we used missing fundamental sounds as stimuli, it is natural to ask whether the F0 and H2 components of the recorded FFRs may be generated peripherally at the level of auditory nerve or even cochlea, reflecting not central representation of sounds but non-linear cochlear compression. We do not believe this is the case. First, our exclusion of the first three harmonics from the stimuli helps reduce the interference of CM and the response at the auditory nerve related to CM. Second, for the F0 components of FFRs to monaural C4 and E4, spectral strength and inter-trial phase coherence at the electrodes near the ears are clearly larger at the contralateral side than at the ipsilateral side (Figure 5). Third, there is hardly any strong connection between electrodes near the ears, e.g., T7/TP7 and T8/TP8, for binaural conditions (Figure 6A). Therefore, the FFRs reported here were not generated at the peripheral level out of cochlear non-linearity.

Then, we showed that the FFRs in response to sounds with different F0s do not reflect activity from a single configuration of neuron ensembles. Both F0 and H2 components of monaurally evoked FFRs, as well as the F0 component of binaurally evoked FFRs, have spatial complexity varying with stimulus F0, although the spatial complexity of the H2 component of binaurally evoked FFRs does not significantly differ across stimulus F0s (Figure 4). In addition, scalp distributions of spectral strength, phase coherence, and temporal peaks of both F0 and H2 components of the FFR vary with stimulus F0 (Figure 5, 7B). Moreover, the pattern of functional connectivity varies with stimulus F0, at least for both F0 and H2 components of monaurally evoked FFRs (Figure 6). If the FFR source configuration were not varying with stimulus F0, then we should not have observed different FFR features across the three tones. Therefore, our findings support that the source configuration for either F0 or H2 component of the FFR is sensitive to stimulus F0.

Our findings confirm that FFR source configuration varies with stimulus frequency, for both F0 and H2 components. Previous literature documents that FFRs in response to frequencies low enough for cortical activity to be included vary in the balance of contributions from high-level (cortical) and low-level (subcortical) structures with stimulus frequency (Bidelman, 2018). Extending to FFRs in response to higher frequencies, our data suggest FFRs with only subcortical contributions also vary in the configuration of neural generators with stimulus frequency. Specifically, we have revealed different spatial complexity for FFRs to different frequencies. Surprisingly, at frequencies like 330 Hz (E4), the spatial complexity is considerably low even for the F0 component of monaural FFRs, indicating the underlying sources are highly consistent in their dipole orientation. From our perspective, it would be helpful to find out all such frequencies in future investigations, as a low spatial complexity (i.e., high inter-channel phase consistency) ensures sufficiently similar phase of FFRs recorded from different recording channels. This helps to reduce the effect of recording channel choice and to facilitate comparisons of data and results across studies and/or labs, particularly those using FFRs recorded with a single channel, which is quite common (Krishnan, 2007; Skoe and Kraus, 2010). Another interesting finding is that the H2 component, at least for monaurally evoked FFRs, also has a varying source configuration, though the subcortical origination of frequencies in this range (>500 Hz in this study) has almost never been challenged. This suggests that the effect of stimulus frequency on FFR source configuration may apply to a relatively wide range of frequency components. However, whether this effect may occur for higher harmonic components (≥H3) requires further investigations.

Moreover, our discoveries support interaction effects of recording channels and stimulation side with stimulus frequency on FFR source configuration, reminding future studies of choosing recording channels and stimulation side with more caution. First, our discoveries brought about reconsideration of an assumption that the same recording channel picks up activity from a fixed configuration of neuron ensembles regardless of stimulus frequency, common in studies using various stimulus frequencies to elicit FFRs (e.g., Hoormann et al., 1992; Bidelman et al., 2011; Skoe and Kraus, 2012; Marmel et al., 2013). However, since the scalp distribution of both spectral strength and phase coherence varies with stimulus frequency, what a fixed recording channel picks up may conceivably alter. For example, vertical recording channels (e.g., Cz to earlobe) are considered to be optimal for collection of upper brainstem activity (Stillman et al., 1978; Galbraith, 1994) but may still pick up strong activity from cochlear and/or auditory nerve (Bidelman, 2015). It is conceivable that the balance of contributions from upper brainstem and auditory nerve to the FFR varies with stimulus frequency (King et al., 2016). Similarly, the frequency function of FFR strength may be attributed partly to the variation of the underlying source configuration (Tichko and Skoe, 2017). Therefore, future studies may require additional evaluation of the scalp distribution to examine the possible impact of source configuration change when making comparisons between FFRs to various frequencies recorded in a certain channel, or deriving group delay in the frequency domain from FFRs elicited by several frequencies.

In addition, our systematic evaluation of the spatial complexity (via GFS) of both monaural and binaural FFRs reveals an interaction effect between stimulus frequency and stimulation side on FFR source configuration, particularly for the F0 component. For the F0 component of the FFR to C4, the spatial complexity is extremely high for monaural FFRs but considerably low for binaural ones; for the F0 component of the FFR to higher F0s, the spatial complexity is low for both monaural and binaural FFRs. This suggests more complicated source configuration for monaural FFRs to low stimulus F0s than for binaural ones. In fact, the difference in source configuration between monaural and binaural FFRs are not fully examined in previous investigations of FFR sources (use only binaural stimulation: Smith et al., 1975; Bidelman, 2015, 2018; Coffey et al., 2016; use only monaural stimulation: Sohmer et al., 1977; Gardi et al., 1979; Zhang and Gong, 2017). From the scalp distributions of FFR spectral strength and phase coherence, it is easily seen that the source configurations underlying monaurally and binaurally FFRs are clearly different for either frequency components and for any of the three tones (Figure 5); monaurally evoked FFRs tend to have a lateralized set of sources, while binaurally evoked FFRs seem to have a symmetric set.

We hypothesize that the variation of FFR source configuration with stimulus frequency may result from different relative contributions of subcortical nuclei to the FFR, at least for binaurally evoked FFRs. Our discovery of the resemblance between FFR and click-ABR topographies provides positive evidence.

As supported by topographic comparison between click-ABR and FFR, we speculate that the F0 component of FFRs in response to sounds with higher F0s might originate from a source configuration dominated by neuron ensembles at lower levels, at least when stimuli were binaurally presented. We used a bootstrap-based statistic to reflect the topographic similarity between click-ABR and FFR, as well as the individual latency when the click-ABR was most topographically correlated to the FFR. The results of the two approaches are consistent with each other in binaural conditions. For the F0 component, the time course of the similarity statistic reaches its peak first for G4, then for E4, and last for C4 (Figure 7B). Similarly, the individual latency when FFR and click-ABR are most topographically correlated is earliest for G4, later for E4, and latest for C4 (Figure 7C). These results suggest that the F0 component of binaurally evoked FFRs in response to stimuli at lower F0s might reflect phase-locked activity dominated by neuron ensembles at higher levels of the auditory brainstem. Consistently, the functional connectivity pattern underlying the F0 component of the FFR is ipsilaterally lateralized for the highest stimulus F0 (393 Hz; G4), contralaterally lateralized for a lower stimulus F0 (330 Hz; E4), and symmetrical for the lowest stimulus F0 (262 Hz; C4), in line with the activation order of various nuclei along the auditory pathway (Figure 6; Marsh et al., 1974).

On the other hand, for the H2 component, the time course of the similarity statistic reaches its peak at similar time points for C4, E4, and G4 (Figure 7B), and the individual latency when FFR and click-ABR are most topographically correlated is also similar across the three tones (Figure 7C). These results lead us to speculate that the H2 component of binaurally evoked FFRs might reflect phase-locked activity dominated by neurons at neighboring locations in the auditory brainstem. In most monaural conditions, the topographic similarity statistic and the individual latency are not consistent with each other, perhaps because the neural sources underlying binaurally evoked FFRs are more focal and thus more akin to the sources of the click-ABR than those of monaurally evoked FFRs. This is true at least for the F0 component, supported by the lower spatial complexity for binaural stimulations (Figure 4).

The possible origins that dominate the binaurally evoked FFR were speculated via comparison with the click-ABR. For the F0 component, the latency when the FFR and the click-ABR are most topographically correlated is comparable to the latency of wave VI for C4, wave V for E4, and earlier than wave V but later than the latency of wave III for G4 (Figure 7C). Therefore, the F0 component might be largely attributed to IC, the site of generation of wave VI, for C4, to SOC or LL, the site of generation of wave V, for E4, and to some place below SOC but above CN, the site of generation of wave III, for G4 (for the generation sites of various waves of the click-ABR, see Møller, 1998; Parkkonen et al., 2009). For the H2 component, the latency of highest topographic correlation between FFR and click-ABR is comparable to that of wave V for all three tones (Figure 7C), suggesting that the H2 component might be largely attributed to SOC or LL regardless of stimulus F0.

The topographic comparison between FFR and click-ABR may be confounded by a few factors. First of all, we did not control the stimulation rate between click-ABR and FFR, but we do not believe that the comparison between FFR and click-ABR is largely confounded. First, we used a presentation rate for the click-ABR that is typical of most clinical protocols (Picton, 2011); rates significantly above (>30 Hz) result in prolonged latencies and diminished waveform morphology (Suzuki et al., 1986), and rates below this lead to impractically long test times when a high number of trials is desired. Second, the topographies of our click-ABRs are consistent with previous reports despite different stimulation rates (Grandori, 1986; Norrix and Glattke, 1996; they used a rate of 21 s^-1^), suggesting that ABR topographies are probably stable unless the stimulation rate dramatically alters. Third, the stimulation rate does not affect the relative order of the latencies of various ABR waves (Rowe, 1978; Suzuki et al., 1986), and thus it does not affect the relative order of the timings at which FFR components are most topographically correlated to the click-ABR.

Another factor that may confound the comparison is the differences between the neural mechanisms that underlie the click-ABR and the FFR, representing transient and sustained activity, respectively. We believe that the resemblance between FFR and click-ABR topographies reported here may be confounded to little extent by the mechanism differences of the two responses. The aim of the topographic comparison is to find time points of topographic alignment between FFRs to different frequencies and individual peaks of the click-ABR. This analysis helps to reveal the timing of the predominant generators of the FFR to different frequencies, but it should be noted that a common timing between the click-ABR and FFR does not necessarily imply that mechanisms generating the click-ABR and FFR are identical. Although it has been shown that the periodicity of the FFR in response to click trains is not simply the superimposition of delayed repeated click-ABRs (Bidelman, 2015), the limited spatial resolution of EEG, together with the relatively small number of recording channels in this study, is likely to impede distinctions between two topographies arising from spatially close neuron ensembles in the same structure. After all, for two sets of topographies to have overlapping generators, their scalp distributions must resemble each other, but resemblance alone is not sufficient for concluding that the neural generators are the same. Future investigations may try high-density EEG recordings to improve the precision of the topographic comparison between FFR and click-ABR.

Third, our use of mandarin Chinese speakers as subjects may need to be noticed when one generalizes the conclusions of this study. It is well-known that speaking a tonal language has long-term effects on the amplitude and the pitch tracking of the FFR (e.g., Krishnan et al., 2005; Bidelman et al., 2011). However, it remains to be investigated whether the effect of linguistic experience is large to the extent that tonal language speakers have a significantly different source configuration of the FFR from non-tonal language speakers.

Despite our observations revealing the variation of FFR source configuration with stimulus frequency, the topographic resemblance between binaural FFR and click-ABR is not sufficient for one to draw conclusions on the specific generating sites of the FFR. Further investigations are necessary to examine our speculation that the variation of FFR source configuration with stimulus frequency is attributed to different relative contributions of subcortical nuclei. If this speculation is true, then the FFR to a certain frequency could be used to separately investigate neural activity of a certain subcortical nucleus and thus the FFR would play a more important role in research or clinical use, additional to neuroimaging approaches that remain limited in their ability to discriminate among subcortical nuclei.

## Conclusion

In summary, we found that the spatial complexity measure, the scalp distribution of spectral strength and phase coherence, and the functional connectivity patterns of FFRs systematically changed with stimulus frequency. Topographic comparisons between FFRs and click-ABRs showed that the F0 component of FFRs to higher frequencies may reflect neural activity at lower levels, but the H2 component may arise at similar levels regardless of stimulus frequency, at least for binaurally evoked FFRs. These pieces of evidence leads us to the idea of a varying FFR source configuration as a function of stimulus frequency, even when only subcortical nuclei contribute to the FFR at higher frequencies above 200 Hz.

## Data Availability

The datasets generated for this study are available on request.

## Author Contributions

QG conceived the study. XZ designed the experiments and collected the data. XZ and QG analyzed the data and wrote the first draft of the manuscript. Both authors contributed to manuscript revision, read and approved the submitted version.

## Conflict of Interest Statement

The authors declare that the research was conducted in the absence of any commercial or financial relationships that could be construed as a potential conflict of interest.
